# Schizophrenia symptoms and functioning in patients receiving long-term treatment with olanzapine long-acting injection formulation: a pooled analysis

**DOI:** 10.1186/1471-244X-12-130

**Published:** 2012-08-31

**Authors:** Joseph Peuskens, Vibeke Porsdal, Jan Pecenak, Peter Handest, Yulia D'yachkova, Radim Brousil, Walter Deberdt

**Affiliations:** 1 University Leuven, Kortenberg, Belgium; 2 Eli Lilly Europe, Herlev, Denmark; 3 Comenius University, Bratislava, Slovakia; 4 University of Copenhagen, Copenhagen, Denmark; 5 Eli Lilly, Vienna, Austria; 6 Eli Lilly, Prague, Czech Republic; 7 Eli Lilly, Brussels, Belgium

## Abstract

**Background:**

This analysis of pooled data evaluates treatment outcomes of patients with schizophrenia receiving maintenance treatment with olanzapine long-acting injection (OLAI) by means of a categorical approach addressing the symptomatic and functional status of patients at different times.

**Methods:**

Patients were grouped into 5 categories at baseline, 6 months, and 12 months. Shifts between categories were assessed for individual patients and factors associated with improvement were analyzed. 1182 patients from 3 clinical trials were included in the current analysis.

**Results:**

At baseline, 434 (36.8%) patients had minimal Positive and Negative Syndrome Scale (PANSS) symptoms but seriously impaired Heinrich Carpenter’s Quality of Life Scale (QLS) functioning; 303 (25.6%) had moderate to severe symptoms and seriously impaired function; 208 (17.6%) had mild to moderate symptoms but good functioning, and 162 (13.7%) had minimal symptoms and good functioning. Baseline category was significantly associated with Clinical Global Impression – Severity (CGI-S), extrapyramidal symptoms, working status, age, and number of previous episodes. The majority of all patients starting OLAI treatment maintained or improved (62% at 6 months and 52% at 12 months) their symptom and functioning levels on OLAI maintenance treatment. Less than 8% of the patients showed worsening of symptoms or functioning. An improvement in category was associated with high PANSS positive and low CGI-S scores at baseline.

**Conclusions:**

We present evidence that a composite assessment of schizophrenic patients including symptom severity and functioning is helpful in the evaluation of maintenance treatment outcomes. This approach could also be useful for the assessment of treatment options in clinical practice.

The trials from which data are reported here were registered on clinicaltrials.gov as NCT00088491, NCT00088465, and NCT00320489.

## Background

Treatment of schizophrenia is a long-term effort, not only aiming to control symptoms but also to maintain or improve functioning, allowing patients to participate in paid employment and other societal activities. Since poor treatment adherence is a major risk for relapse, the use of long-acting injection (“depot”) formulations of antipsychotics should have advantages in maintenance treatment of patients with schizophrenia with a history of non-compliance and relapse [1-3].

In recent years, the atypical antipsychotics risperidone, paliperidone, and olanzapine became available as depot formulations allowing effective long-acting antipsychotic treatment while avoiding some of the side effects of the earlier available depot formulations of typical antipsychotics.

The present pooled analysis assesses the overall development of the symptomatic and functional status of patients with schizophrenia observed during 3 clinical studies with the olanzapine long-acting injection (OLAI) formulation. In all studies, maintenance treatment outcomes of schizophrenia symptoms were measured by the positive and negative Syndrome scale (PANSS), and functioning by the Heinrich Carpenter’s quality of life scale (QLS). In this scale, functioning is assessed in particular with the QLS Interpersonal Relations and QLS Instrumental Role domains. Analyses were performed on the data collected at baseline and after 6 and 12 months of patient participation in these studies with OLAI.

A comprehensive description of the status of patients with schizophrenia should include an assessment of schizophrenia symptoms and of patient functioning. Consequently, the present analysis is based on a classification of patients into 5 distinct clusters or categories (A to E) that were previously identified in a population of patients with schizophrenia treated over approximately 30 weeks with oral antipsychotics [4]. This cluster analysis by Lipkovich et al. also provided the categorization rules, based on a PANSS 5-factor model [4] and QLS scores, allowing a categorical classification of patients with high specificity and sensitivity. Changes in PANSS and QLS scores over time may result in a change in category for a particular patient.

The objective of the analyses reported is to evaluate how non-acute schizophrenia patients with different baseline status develop in terms of their symptoms and functioning during maintenance treatment as analyzed by changes in their assignment to the pre-defined categories consisting of a combination of symptomatic status and level of functioning. Further, factors associated with categorical improvement in symptoms and/or functioning are identified through statistical modeling. As an alternative approach to the cluster analysis approach, a remission analysis based on the Andreasen remission criteria was performed [5]. Our results may add important information about factors associated with long-term benefits for patients and society when patients are receiving maintenance treatment with depot formulations of olanzapine.

## Methods

### Datasets

The present analysis was performed on data from patients with schizophrenia who were not acutely ill and were receiving maintenance treatment with OLAI medication. These patients were enrolled in 3 clinical trials: HGKA, HGLQ, and HGKB. Patients in HGKB could have previously participated in studies LOBS, HGKA, and HGJZ. All 5 studies were sponsored by Eli Lilly & Company. The studies used in the analyses had measurements of PANSS and of QLS available at baseline, and at 6 months (± 1 month). PANSS and QLS data were also evaluated at 12 ± 3 months (1 year). All patients gave written informed consent prior to participation in any of the studies. The main study design features of the studies included in the pooled analysis are summarized below:

Study HGKA was a 6 month, double-blind, randomized, multicenter study on 1065 clinically stable outpatients. 753 completed the reporting interval [6]. Of those, 599 patients with OLAI dose of 300 mg/2 weeks (n = 140), 405 mg/4 weeks (n = 314), or 150 mg/2 weeks (n = 140) were included in the analysis. Patients on oral olanzapine as well as on OLAI at the very low dose of 45 mg/2 weeks (given in lieu of placebo) were not included in the present analysis.

Study HGLQ was a 2 year, open-label, randomized, multicenter study comparing the long-term treatment effectiveness and safety of OLAI with oral olanzapine. 261 patients who received an injection of 405 mg OLAI at baseline followed by individual dosing of 150 mg to 405 mg/4 weeks were included in the analysis [7].

Study HGKB was an open label extension safety study offered to patients who completed or discontinued from study HGKA, study HGJZ (investigating superiority of OLAI vs. placebo), or study LOBS (investigating product performance and bioavailability of OLAI). The initial dose of OLAI was 210 mg with a second injection after 2 weeks (150–405 mg). Subsequently, individual dosing with dosing intervals of 2 to 4 weeks and a maximum cumulative total dose of 600 mg during a 4-week period was allowed [8].

All study protocols were approved by institutional review boards at each site.

Data collected during the acute treatment periods in the short term studies HGJZ and LOBS were not included in the present analysis because these patients were either acutely ill (HGJZ) or received an OLAI dosage schedule specifically for pharmacokinetic evaluations (LOBS). Therefore, these data were not helpful for assessing maintenance treatment effects of OLAI. For the present analysis, baseline for patients from these studies was defined as entry into study HGKB. Baseline for patients in HGKA and HGLQ was the first assessment in these studies.

The following baseline variables collected in all three studies were used for statistical modeling: age, baseline category, baseline remission status, extrapyramidal symptoms (EPS), geographic region, CGI-S score , number of previous episodes, PANSS total and subscores (anxiety/depression, disorganized, hostility, negative, positive), QLS total and domains (interpersonal relations, instrumental role functioning), study indicator (HGKA, HGLQ, HGKB), gender, time since diagnosis, weight, and employment status (unemployed, working, retired).

Overall data from 1182 patients at baseline, 864 patients at 6 months and 687 patients at 12 months were used for the present analysis.

### Outcome measures

Psychiatric symptoms were assessed using the PANSS [9]. In the present study, for creating the patient categories, we followed the PANSS factor analysis by Marder et al. [10]. The 5 main factor scores were: 1) PANSS Negative factor (PANSS NEG; 7 items: 8–11,13,21,30); 2) PANSS Positive factor (PANSS POS; 8 items: 1,3,5,6,14,15,23,26); 3) PANSS Disorganized factor (PANSS DIS; 7 items: 2,12,19,24,25,27,29); 4) PANSS Hostility factor (PANSS HOS; 4 items: 4,7,22,28); and 5) PANSS Anxiety/Depression factor (PANSS DEP; 4 items: 16,17,18,20). Scores on individual PANSS items ranged from 1 (no symptoms) to 7 (severe symptomatology). PANSS factor scores were divided by the number of items per factor (i.e. normalized factor scores range from 1 to 7).

Functioning in all studies was assessed with the QLS total score. The QLS items range from 0 (worst) to 6 (best). For the analysis we used the 2 QLS domains (interpersonal relations and instrumental role [11]) as categorizers identified in the Lipkovich et al [4] cluster analysis.

QLS Interpersonal Relations domain measuring the qualitative aspects of interpersonal relationships; consisting of 8 items, and

QLS Instrumental Role domain measuring the level of and the satisfaction with occupational role functioning; consisting of 4 items.

These 2 domains of the QLS represent the part of the QLS that reflects the functional aspects of a patient's quality of life including the patient's ability to work and to maintain relationships with others. Overall scores for domains as well as the total score were divided by the number of the respective items.

Based on cluster analysis reported in Lipkovich et al [4], patients with schizophrenia can be grouped into 5 different clusters (categories) based on severity of their clinical symptoms as measured by PANSS and on the level of their functioning, as measured by the QLS Interpersonal and QLS Instrumental domains. For the current analysis, the classification rules of this previous publication were used. The category cutoffs used, as determined by a classification tree algorithm [CART, [12]], were based on the QLS Instrumental domain score and on the PANSS DIS and PANSS POS subscores. These decision rules do not yield mutually exclusive categories but are able to separate categories with high specificity between 87% and 98%.

The categories contain patients with good symptom control and moderate to good functioning (Category A), patients with either good symptom control and impaired functioning (Category B) or insufficient symptom control and moderate to good functioning (Category C), and the most severe categories include patients with moderate to severe symptoms and impaired functioning (Category D) and with severe psychiatric symptoms and impaired functioning (Category E).

Categorical improvement of a patient was defined as moving from Categories B or C to Category A, or from Categories D or E to Categories A, B, or C. Thus, patients in Category A could not improve and were excluded from the analysis of improvement. Worsening of a patient was defined as moving from Category A to Categories B, C, D, or E or from Categories B or C to Categories D or E. As only few patients were categorized in Categories D and E both categories were combined and could not worsen. Thus, they were excluded from the analysis of worsening.

As an additional measure of long term treatment outcomes in addition to the cluster analysis described above, the proportion of patients in remission and sustained remission were analyzed. Remission was defined based on the Andreasen criteria [5]. Sustained remission was defined as maintaining simultaneous ratings of “mild” or better (score of 1, 2, or 3) over a 6 month period on the following items of the PANSS: delusions, conceptual disorganization, hallucinatory behavior, mannerisms/posturing, unusual thought content, blunted affect, social withdrawal, and lack of spontaneity.

Cross-sectional remission at baseline or any other specific time point was defined similarly, but excluded the requirement for maintenance over a 6 month period.

### Statistical procedure

Patient baseline and post baseline characteristics were summarized using descriptive statistics and compared among baseline categories using analysis of variance (ANOVA) for continuous variables and chi-square test for categorical variables. Reasons for discontinuation and baseline characteristics of patients remaining in the analyses versus those discontinuing within 6 months were summarized and compared using t-test for continuous variables and chi-square test for categorical variables.

Baseline factors were assessed for their association with categorical improvement using stepwise logistic regression. These analyses were performed for categorical improvements from baseline to 6 months and to 12 months (see definition of categorical improvement in 'Outcome measures' section).

At the first step of modeling reported in Table 1 under the ‘Single variable model’ heading, a number of single factor logistic regression models were fitted. Each model evaluated a single baseline variable in relation to the outcome (categorical improvement) without adjustment for other variables. The following baseline variables were used as potential predictors: age, baseline category, baseline remission status, extrapyramidal symptoms (EPS), geographic region, Clinical Global Impression Severity (CGI-S) score [13], number of previous episodes, PANSS total and subscores (anxiety/depression, disorganized, hostility, negative, positive), QLS total and domains (interpersonal relations, instrumental role functioning), study indicator (HGKA, HGLQ, HGKB), gender, time since diagnosis, weight, and employment status (unemployed, working, retired). This approach allowed for screening of a high number of correlated variables and provided additional information about potential association prior to adjustment for other variables. SAS automatic procedures would exclude all patients who have missing values for at least one of the variables and have convergence issues with correlated variables, and therefore were not used. The number of covariates entering at the second step in the multiple logistic regression model was reduced by manual variable selection based on the first step modeling results to avoid over-fitting.

**Table 1:** Baseline predictors associated with categorical improvement after 6 Months and 12 Months of treatment with OLAI

	**Logistic regression statistics**		
**Model**	***Odds ratio***	***95% CI of Odds ratio***	***p-value (for effect)***
***Single variable model 6 Months***			
Age [1 year]	0.989	0.975; 1.002	0.0937
Time since diagnosis [1 year]	0.979	0.959; 0.998	0.0327
Working status			
unemployed vs. retired	1.023	0.620; 1.688	0.9287
working vs. retired	1.564	0.925; 2.644	0.0948
Clinical Study			
HGKA vs. HGKB	1.890	1.262; 2.830	0.0020
HGLQ vs. HGKB	0.972	0.603; 1.566	0.9068
** Baseline category**			
** C vs. B**	**3.007**	**1.966; 4.600**	**<0.0001**
** D vs. B**	**6.656**	**4.386; 10.099**	**<0.0001**
** E vs. B**	**3.043**	**1.585; 5.841**	**0.0008**
CGI-S [1 point higher at baseline]	0.787	0.659; 0.941	0.0084
** PANSS total [1 point higher at baseline]**	**1.010**	**1.000; 1.020**	**0.0622**
PANSS positive	1.553	1.210; 1.992	0.0005
PANSS anxiety/depression	1.326	1.066; 1.649	0.0111
PANSS hostility	1.253	0.992; 1.583	0.0586
** QLS total [1 point higher at baseline]**	**1.135**	**1.035; 1.245**	**0.0072**
QLS Instrumental role funct.	1.151	1.026; 1.291	0.0162
QLS Interpersonal relations	1.159	1.008; 1.333	0.0381
** Remission baseline [yes vs, no]**	**1.061**	**0.778; 1.449**	**0.7078**
** Remission 6 months [yes vs. no]**	**2.420**	**1.670; 3.507**	**<0.0001**
***Stepwise multivariate logistic regression 6 Months***			
PANSS positive	2.840	1.834; 4.399	<0.0001
CGI-S	0.619	0.463; 0.828	0.0012
***Single variable model 12 Months***			
Clinical Study			
HGKA vs. HGKB	1.592	1.048; 2.418	0.0294
HGLQ vs. HGKB	0.905	0.562; 1.458	0.6811
** Baseline category**			
** C vs. B**	**3.316**	**2.085; 5.271**	**<0.0001**
** D vs. B**	**6.936**	**4.351; 11.057**	**<0.0001**
** E vs. B**	**5.879**	**2.742; 12.605**	**<0.0001**
** PANSS total**	**1.018**	**1.006; 1.029**	**0.0024**
PANSS positive	1.798	1.354; 2.387	<0.0001
PANSS anxiety/depression	1.370	1.066; 1.761	0.0141
PANSS disorganized	1.390	1.062; 1.821	0.0167
PANSS hostility	1.565	1.193; 2.053	0.0012
** QLS total**	**1.157**	**1.045; 1.281**	**0.0049**
QLS Instrumental role funct.	1.178	1.038; 1.337	0.0112
QLS Interpersonal relations	1.167	0.999; 1.363	0.0520
** Remission baseline**	**1.013**	**0.720; 1.426**	**0.9391**
** Remission 12 months**	**2.628**	**1.710; 4.039**	**<0.0001**
***Stepwise multivariate logistic regression 12 Months***			
PANSS positive	1.537	1.129; 2.093	0.0063

In total, 22 variables were assessed in the single variable logistic regression models. Variables in bold were identified as potentially overlapping variables and were not included in the stepwise multivariate logistic regression model. *CGI-S*: Clinical Global Impression – Severity, *CI*: confidence interval, *PANSS*: Positive and Negative Symptoms Score, *QLS*: Heinrich Carpenter’s Quality of Life Scale.

At the second step of modeling, reported in Table 1 under the ‘Stepwise multivariate logistic regression’ heading all baseline variables that were identified as potentially significant (p < 0.1 in the single factor model) were simultaneously included in a multivariate model for stepwise selection of covariates based on significance cut-off of p < 0.1 entry into the combined model. After the stepwise procedure only those variables that were independently predicting outcome remained in the model. Other variables were excluded due to co-dependencies, even if they were significant as a single predictor. To avoid “overlap” in predictors and issues with co-linearity, variables derived from predictors already included in the model (total scores for the PANSS and QLS scales, remission status, and baseline category) were not used in the multiple regression model. No imputation of missing data was used. SAS version 9.1.3 (SAS Institute, Cary, N.C.) was used for all analyses.

## Results

### Baseline categories and dropouts at 6 months

1182 patients with baseline PANSS and QLS data available were included in the current analysis. Patients included in the analysis were 66.8% males, had a mean age of 39.6 years (range: 18 to 74 years), and 46.5% were from the Americas, 44.8% from Europe and 8.6% from other areas. Baseline mean PANSS total score was 62.1 (±16.4), and mean QLS total score was 1.65 (±1.81). EPS were reported for 13.9% of the patients.

339 patients (28.7%) discontinued from one of the 3 respective studies during the first 6 months. The retention rate by study was 75.3% (HGKA, 451/599); 73.6% (HGLQ, 192/261), and 69.5% (HGKB, 221/318). Of all 864 patients with data at 6 months (i.e. 21 patients discontinuing at the 6 months visit had still data available), 787 (91.1%) had sufficient data available for the assessment of categorical changes in schizophrenia symptoms (PANSS) and health-related functioning (QLS).

The most common (>10% of all discontinuations) reasons for discontinuations in the first 6 months were subject decision (129, 38.1%), adverse event (56, 16.5%), lack of efficacy (41, 12.1%), and lost to follow-up (38, 11.2%).

We have compared baseline characteristics of patients who remained for at least 6 months in the studies with those who discontinued (Table 2). Patients discontinuing early had more severe psychiatric symptoms at baseline – in particular acute symptoms as measured by the PANSS POS, PANSS HOS, and PANSS DEP scores. Higher rates were also found for other baseline measurements, reflecting decreased functioning such as CGI-S, working status, baseline remission, and QLS instrumental role functioning. No significant differences between dropouts and patients remaining in the studies were seen for age, geographic region, gender, PANSS NEG and PANSS DIS scores, QLS interpersonal relations domain, EPS at baseline, and number of previous episodes.

**Table 2:** Baseline characteristics for all patients and for patients remained vs. patients who discontinued within 6 months from baseline

	**Patients n (%)**			
**Characteristic (%)**	***Remained (N = 843)***	***Discontinued (N = 339)***	***Total (N = 1182)***	**p-value**
Geographic Region				0.129
Western Europe	148 (68.8)	67 (31.2)	215	
Eastern Europe	237 (75.2)	78 (24.8)	315	
Americas	380 (69.1)	170 (30.9)	550	
Other	78 (76.5)	24 (23.5)	102	
Gender				0.391
Female	274 (69.7)	119 (30.3)	393	
Male	569 (72.1)	220 (27.9)	789	
EPS				0.180
No	542 (74.0)	190 (26.0)	732	
Yes	113 (68.9)	51 (31.1)	164	
Working Status				0.003
Unemployed disab.	349 (70.2)	148 (29.8)	497	
Paid work	181 (81.2)	42 (18.8)	223	
Retired	101 (82.1)	22 (17.9)	123	
Keeping house	73 (70.2)	31 (29.8)	104	
Unemployed unrel.	61 (81.3)	14 (18.7)	75	
Student	33 (89.2)	4 (10.8)	37	
Volunteer	23 (69.7)	10 (30.3)	33	
Self-employed	15 (78.9)	4 ( 21.1)	19	
Baseline Remission				
Cross-sectional	455 (74.6)	155 (25.4)	610	0.010
**Mean (SD)**				
Age [year]	39.4 (11.43)	40.1 (10.89)	39.6 (11.28)	0.293
***PANSS Total***	61.4 (16.20)	64.1 (16.66)	62.1 (16.37)	0.012
PANSS POS	2.1 (0.66)	2.3 (0.77)	2.1 (0.70)	<0.001
PANSS NEG	2.4 (0.83)	2.4 (0.83)	2.4 (0.83)	0.969
PANSS DIS	2.1 (0.66)	2.2 (0.68)	2.1 (0.67)	0.274
PANSS HOS	1.5 (0.62)	1.7 (0.70)	1.6 (0.65)	0.003
PANSS DEP	1.9 (0.70)	2.0 (0.71)	1.9 (0.70)	0.015
**QLS Total**	1.8 (1.84)	1.4 (1.73)	1.7 (1.81)	0.001
QLS Interpersonal relations	2.8 (1.19)	2.7 (1.19)	2.8 (1.19)	0.074
QLS Instrumental role funct	2.5 (1.49)	2.2 (1.45)	2.4 (1.49)	<0.001
CGI-S	3.0 (0.93)	3.3 (0.97)	3.1 (0.95)	<0.001
No. of previous episodes	1.4 (1.53)	1.8 (2.74)	1.5 (1.91)	0.121

*CGI-S*: Clinical Global Impression – Severity, *EPS*: extrapyramidal symptoms, *PANSS*: Positive and Negative Symptoms Score, *QLS*: Heinrich Carpenter’s Quality of Life Scale, *SD*: standard deviation.

**Figure 1:**
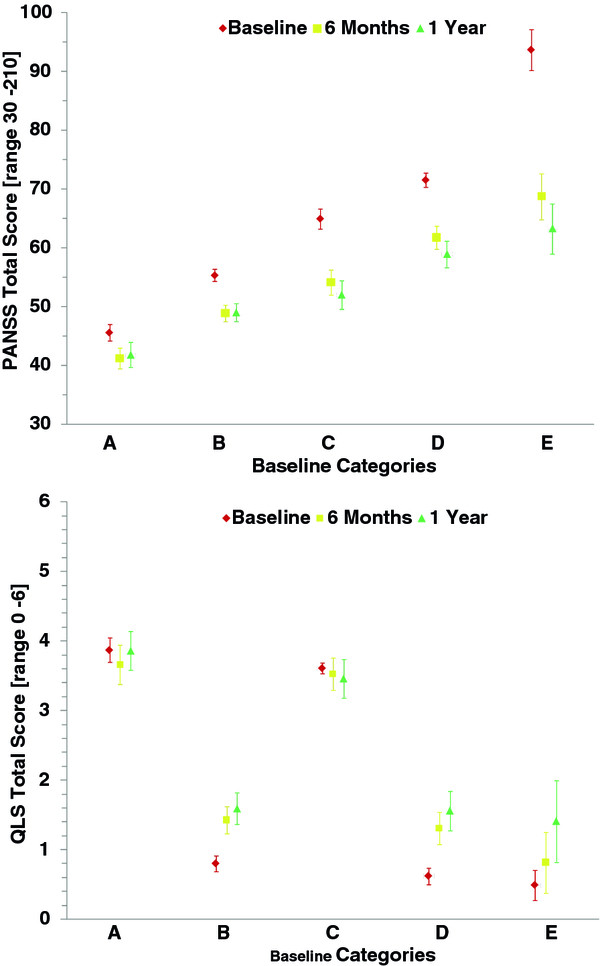
Mean (95% CI) PANSS and QLS total score over time by baseline category.

Table 3 shows baseline characteristics in relationship to baseline categories (PANSS and QLS scores at baseline are reported in Table 4 and Figure 1).

**Table 3:** Patient baseline characteristics by category at baseline

	**Categories n (%)**					
**Characteristic (%)**	***A N = 162***	***B N = 434***	***C N = 208***	***D N = 303***	***E N = 72***	**p-value**
Study						0.068
HGLQ	32 (19.8)	123 (28.3)	38 (18.3)	68 (22.4)	0	
HGKA	82 (50.6)	210 (48.4)	122 (58.7)	142 (46.9)	40 (55.6)	
HGKB	48 (29.6)	101 (23.3)	48 (23.1)	93 (30.7)	32 (44.4)	
Geographic Region						0.002
Western Europe	24 (14.8)	79 (18.2)	41 (19.7)	60 (19.8)	10 (13.9)	
Eastern Europe	53 (32.7)	115 (26.5)	55 (26.4)	72 (23.8)	18 (25.0)	
Americas	60 (37.0)	210 (48.4)	79 (38.0)	160 (52.8)	41 (56.9)	
Other	25 (15.4)	30 (6.9)	33 (15.9)	11 (3.6)	3 (4.2)	
Gender						0.491
Female	59 (36.4)	145 (33.4)	69 (33.2)	93 (30.7)	25 (34.7)	
Male	103 (63.6)	289 (66.6)	139 (66.8)	210 (69.3)	47 (65.3)	
EPS						0.013
Yes	12 (7.4)	58 (13.4)	30 (14.4)	48 (15.8)	16 (22.2)	
Working Status^1^						<0.001
Paid work	72 (44.4)	50 (11.5)	70 (33.7)	28 (9.2)	3 (4.2)	
Retired	19 (11.7)	61 (14.1)	15 (7.2)	23 (7.6)	4 (5.6)	
Unemployed dis.	24 (14.8)	194 (44.7)	54 (26.0)	177 (58.4)	46 (63.9)	
Cross-sectional remiss.						<0.001
No	25 (15.4)	168 (38.7)	89 (42.8)	216 (71.3)	71 (98.6)	
Yes	137 (84.6)	266 (61.3)	119 (57.2)	87 (28.7)	1 (1.4)	
**Mean (SD)**						
Age [year]	37.6 (10.6)	40.7 (11.6)	37.9 (11.1)	40.0 (11.2)	40.1 (11.3)	0.002
CGI-S	2.34 (0.85)	2.96 (0.89)	3.06 (0.84)	3.56 (0.76)	4.01 (0.84)	*<0.001*
No. of previous episodes	1.07 (1.25)	1.60 (2.50)	1.34 (1.31)	1.54 (1.68)	1.85 (1.75)	0.017

^1^ Only the 3 largest categories (>10% of all patients) are shown as working status; 6 additional categories were defined and were included in the statistical analysis (self employed, student, volunteer, keeping house, unemployed unrelated),*CGI-S:* Clinical Global Impression – Severity, *EPS*: Extrapyramidal Symptoms, *SD*: standard deviation.

By applying the rules based on Lipkovich et al. [4] and described in the 'Outcomes measures' section to the baseline scores of patients in this analysis, patients were grouped into the 5 pre-specified categories. The largest category (Category B: n = 434, 36.7%, see Table 4 and Figure 1) consisted of patients with minimal symptoms of schizophrenia according to the PANSS total score and with seriously impaired functioning according to the QLS score, followed by patients with moderate to severe PANSS total score symptoms and seriously impaired function according to QLS (Category D: n = 303, 25.6%). Category C (n = 208, 17.6%) comprised patients with moderate PANSS total scores but good functioning according to QLS, and Category A comprised patients with minimal schizophrenic symptoms and good functioning (n = 162, 13.7%). As only non-acutely ill patients were eligible for participation in these studies, only 72 (6.1%) patients were grouped in Category E with severe schizophrenic symptoms and impaired functioning.

**Table 4:** Patient categories at baseline, 6 months and 12 months, and change of categories at 6 months and 12 months

		**Categories at 6 (±1) months**							
**Baseline category**	**Num. of patients**^**1**^	**A**	**B**	**C**	**D**	**E**	**Missing at 6 months**^**2**^	**Category improved**^**2**^	**Category worsened**^**2**^
**A**	162 (14%)	86	***26***	***7***	***0***	***0***	43 (27%)	N.A.	***33 (20%)***
**B**	434 (37%)	**61**	210	9	***12***	***1***	141 (33%)	**61 (14%)**	***13 (3%)***
**C**	208 (18%)	**68**	16	61	***8***	***1***	54 (26%)	**68 (33%)**	***9 (4%)***
**D**	303 (26%)	**14**	**80**	**18**	62	2	127 (42%)	**112 (37%)**	N.A.
**E**	72 (6%)	**3**	**14**	**3**	18	7	27 (38%)	**20 (28%)**	N.A.
**Missing**	3 (0.3%)	0	0	0	0	0	3		
**Total**	1182 (100%)	232 (20%)	346 (29%)	98 (8%)	100 (8%)	11 (1%)	395 (33%)	**261 (26%)**	***55 (7%)***
		**Categories at 12 (±3) months**							
**Baseline category**	**Num. of patients**^**1**^	**A**	**B**	**C**	**D**	**E**	**Missing at 1 year**^**2**^	**Category improved**^**2**^	**Category worsened**^**2**^
**A**	162 (14%)	83	***22***	***7***	***1***	***0***	49 (30%)	N.A.	***30 (19%)***
**B**	434 (37%)	**58**	169	7	***11***	***4***	185 (43%)	**58 (13%)**	***15 (3%)***
**C**	208 (18%)	**62**	21	36	***3***	***0***	86 (41%)	**62 (30%)**	***3 (1%)***
**D**	303 (26%)	**20**	**60**	**16**	43	1	163 (54%)	**96 (32%)**	N.A.
**E**	72 (6%)	**7**	**12**	**4**	8	4	37 (51%)	**23 (32%)**	N.A.
**Missing**	3 (0.3%)	0	0	0	0	0	3		
**Total**	1182 (100%)	230 (19%)	284 (24%)	70 (6%)	66 (6%)	9 (1%)	523 (44%)	**239 (24%)**	***48 (6%)***

Patients improving from baseline to 6 months/1 year are indicated in bold. Patients worsening from baseline to 6 months/1 year are indicated in bold italics. ^1^% to the column total across baseline categories; ^2^% to the row total within baseline category N.A.: not applicable.

Baseline category was significantly related to CGI-S, presence of baseline EPS, working status, cross-sectional remission, age, and number of previous episodes (Table 3). Some differences regarding geographical regions were found. In particular more patients from Eastern Europe were categorized into Category A. However, this might reflect recruitment differences of patients by studies and is considered as not clinically relevant.

The rate of patients discontinued ranged from 26% to 42% by baseline category at 6 months and from 30% to 54% at 12 months (see Table 4). The lowest rates were seen in Category A and the highest rates in the worst categories (D and E).

### Categorical changes after 6 months and 12 months of treatment

The change of category membership from baseline to the 6 months and 12 months assessments is shown in Table 4 and Figure 1.

Overall at 6 months, 261 (26%) of all patients who could improve in category (all but A) did improve, 471 (40%) of all patients remained in their baseline category, and 55 (7%) of all patients who could get worse (all but D and E) worsened. For 395 (33%) of all patients, a classification into category at 6 months was not possible because they had no PANSS or QLS score corresponding to the 6 month visit available. By baseline category, improvement was seen in >30% of patients in baseline Categories C and D but for less than 15% in baseline Category B. Worsening was seen in about 20% of patients in baseline Category A and was <5% in baseline Categories B and C.

The proportion of patients improving (239; 24%) or worsening (48; 6%) after 12 months of OLAI treatment was similar to those proportions seen at 6 months. The proportion of patients who could not be classified due to either discontinuation or missing data after 12 months was 44% (523).

Table 5 shows the PANSS Total score and QLS Interpersonal and QLS Instrumental domain scores at baseline, 6 months, and 12 months overall and by baseline category and the cross-sectional and sustained remission status assessed at baseline, 6 months, and 12 months.

**Table 5:** PANSS total score, QLS functional domain scores, and remission at baseline and changes at 6 Months and at 12 months

		**Categories**					
**Scores**		***A – (N)=162***	***B – (N)=434***	***C – (N)=208***	***D – (N)=303***	***E – (N)=72***	**Overall – (N) = 1182**
PANSS Total							
Baseline		45.56 (9.22)	55.29 (11.19)	64.91 (12.80)	71.50 (10.74)	93.63 (14.64)	61.53 (16.12)
6 months		−4.39 (8.95)	−6.41 (11.09)	−10.80(12.98)	−9.73 (12.56)	−24.90(21.70)	−8.77 (13.14)
12 months		−3.79 (11.55)	−6.32 (12.14)	−12.94(12.78)	−12.63(13.11)	−30.39(20.72)	−9.75 (14.29)
QLS Interpersonal							
Baseline		3.75 (1.03)	2.48 (1.00)	3.56 (1.02)	2.22 (1.03)	2.00 (0.98)	2.77 (1.19)
6 months		0.02 (0.85)	0.38 (0.91)	0.11 (0.87)	0.41 (0.80)	0.42 (0.85)	0.28 (0.88)
12 months		0.20 (0.89)	0.47 (0.99)	0.03 (0.92)	0.46 (0.82)	0.61 (0.83)	0.34 (0.93)
QLS Instrumental							
Baseline		4.26 (0.73)	1.75 (0.97)	4.11 (0.64)	1.50 (0.94)	1.27 (0.99)	2.53 (1.49)
6 months		−0.34 (1.13)	0.52 (1.16)	−0.10 (0.97)	0.51 (1.13)	0.43 (1.07)	0.26 (1.16)
12 months		−0.19 (1.04)	0.70 (1.27)	−0.10 (1.02)	0.68 (1.25)	1.05 (1.19)	−0.41 (1.25)
**Remission – based on observed cases**							
**n (%)**		***A***	***B***	***C***	***D***	***E***	***Overall***
Baseline - cross-sectional		137 (84.6)	266 (61.3)	119 (57.2)	87 (28.7)	1 (1.4)	610 (51.6)
6 months - cross-sectional		114 (95.8)	235 (80.2)	125 (81.2)	104 (59.1)	15 (33.3)	594 (75.5)
6 months - sustained		99 (83.2)	159 (54.3)	90 (58.4)	46 (26.1)	1 (2.2)	395 (50.2)
12 months - cross-sectional		106 (93.8)	204 (81.9)	101 (82.8)	95 (67.9)	15 (42.9)	521 (79.1)
12 months - sustained		104 (92.0)	191 (76.7)	95 (77.9)	76 (54.3)	10 (28.6)	476 (72.2)

*PANSS:* Positive and Negative Symptoms Score, *QLS*: Heinrich Carpenter’s Quality of Life Scale, *SD*: standard deviation.

Cross-sectional remission status improved in 199 patients (17%) from no remission at baseline to remission at 6 months. Worsening from baseline remission to no remission at 6 months occurred in 29 patients (2%). Remission status sustained at 6 months in 395 (33%) of all patients. Sustained remission after 12 months was achieved by 476 (40%) patients, only 49 (4%) of those patients in cross-sectional remission at baseline did not achieve sustained remission at 12 months.

### Baseline predictors associated with improvement

The single variable logistic regression analyses found a significant influence of a number of baseline covariates on categorical improvement of patients at 6 and 12 months. All variables with a significant influence on categorical improvement (except baseline category) are summarized in Table 1.

Based on these, the stepwise multiple logistic regression analyses were performed for categorical improvement at 6 months and 12 months. The significant predictors identified from the stepwise multiple logistic regression are shown in Table 1. An odds ratio estimate greater than 1 indicates higher chances for improvement. Odds ratios were calculated based on an increase of 1 unit of the baseline value for continuous scales or compared to a reference category at baseline for categorical variables.

The odds for achieving a better category at 6 months and 12 months were better for patients with a higher (worse) baseline PANSS POS. In contrast, 6 month categorical improvement was more probable for patients with a lower (better) baseline CGI-S. All other variables that were significant as single predictors lost their significance in the joint model and were excluded by the stepwise procedure. The shaded variables were not entered in the model due to the fact that they were derived from other variables as described in the statistical procedure section.

## Discussion

The majority of patients who stayed on 6 months maintenance treatment with OLAI improved or at least maintained their symptoms and functioning level as measured by membership in 5 pre-specified categories. Improvements from baseline occurred in all categories where this was possible. Improvements in clinical symptoms were also evident when assessing remission as an alternative efficacy parameter. The proportion of patients in cross-sectional remission increased markedly from baseline to 6 months, and about 3/4 of the patients on OLAI treatment achieved sustained remission according to Andreasen criteria after 12 months [5].

Discontinuation rates in this pooled analysis are difficult to interpret since in some instances the reasons for drop-out were linked to specific characteristics of the studies, e.g. planned study termination after 6 months of treatment in HGKA, randomization to fixed dosing schedules in HGKA, or limitation to a maximum dose of 405 mg/4 weeks in HGLQ. The rate of patients discontinuing before the 6 month analysis was moderate (29%). The variables associated with early discontinuation might suggest a certain bias due to patients with worsening symptoms discontinuing more frequently. However, only a minority of patients discontinued due to lack of efficacy. Discontinuation rates were similar to results from other studies in non-acute schizophrenic patients using long-acting risperidone [14-16] and the majority of patients in the poor baseline categories remained in the study.

The dropout rates encountered in this analysis and the heterogeneity of the analysis population due to the differing designs of the source studies are a limitation of the current analysis, and might have led to a bias by retaining only patients with an acceptable or positive response to OLAI treatment during the course of the studies. Regarding study design important differences are the different durations of pre-treatment at baseline and the fact that HGKA was a double blind study whereas HGLQ and HGKB were open-label studies. Also, no consistent information about concomitant psychosocial treatment was available and the impact of this could not be included in the current analyses. In addition, due to the study design of the source studies no comparison to other antipsychotic treatments was possible and no across-study procedures for maintaining interrater-reliability were performed.

The majority of the patients in our analysis was clinically stable and was classified in Categories A, B, and C. Patients with residual psychotic symptoms were assigned to the unfavorable Categories D and E. Our analysis, looking at outcome at discrete points in time, is not well-suited to evaluate the frequency of acute relapses. However, very few patients worsened to Category E, representing a status of severe psychosis. Generally, the risk of symptom exacerbation in patients initially doing well on OLAI treatment was small.

Logistic regression analyses identified a significant association of PANSS positive symptoms at baseline with categorical improvement after 6 months and 12 months, indicating that patients with acute baseline positive symptoms had better chances to improve over time. CGI-S at baseline was also associated with categorical improvement at 6 months, however with better odds for improvement in patients with low CGI-S at baseline. We suggest that PANSS positive symptoms reflect the current psychotic symptoms of a patient and these may vary strongly over time, depending on the course of disease and in particular treatment compliance, leading to an improvement when effective antipsychotic treatment is continuously provided by the depot formulation. In contrast, CGI-S may measure the general status of the patient as assessed by the physician, who may include historical knowledge about the patient, the disease history, and the overall level of functioning of the patient.

Symptoms at baseline were an important predictive factor for treatment outcomes. Surprisingly, some other factors previously reported as predictors for maintenance treatment outcomes in schizophrenic patients in previous studies could not be corroborated in the present analysis. There were no significant differences found for gender, remission status at baseline, disease history, and age [17-19]. However, there were some differences in the type of patients and follow up duration. For example, Möller et al. [17] observed only patients first hospitalized and over a much longer duration of up to 15 years. Furthermore in a more recent review of Emsley et al. [20] only early treatment response, baseline symptom severity and subjective well-being were mentioned as important known predictive factors for remission in schizophrenia.

Outcomes of schizophrenia treatments can be assessed by many measures in several dimensions. As no single individual measure seems to be sufficient for covering a complex disorder as schizophrenia, there have been efforts to find more comprehensive approaches to measure the effectiveness of treatment and patient outcomes [21,22]. The categories used in the present analysis originate from a statistical approach based on well-established symptomatic (PANSS) and functional (QLS) measures combining 2 main dimensions of schizophrenia. Since these categories were derived from a cluster analysis, they do not refer to arbitrary definitions but provide evidence-based criteria for categorization. This approach provides a more comprehensive measure for treatment outcomes in schizophrenia which deserves further evaluation.

In the present sample, even patients in the best category had not achieved good levels of functioning at baseline. This result reflects the serious impact of schizophrenia on functioning in comparison with other psychotic or non-psychotic disorders. When very strict criteria including functioning are used for definition of recovery, only a small proportion of patients with schizophrenia will be able to achieve this target [23-25].

## Conclusions

Overall, this pooled analysis shows that patients who remain on maintenance treatment with OLAI can improve both their schizophrenic symptoms and their functioning. We have shown that there is a potential to treat and improve patients who are already stable. High levels of acute psychotic symptoms usually discourage clinicians and patient relatives and may lead to a suboptimal use of treatment options in severely ill patients. However, our results show that improvement is possible despite severe symptoms and that intensive treatment (e.g. OLAI with regular follow-up) can lead to a significant improvement for patients. Finally, this analysis provides evidence that a composite assessment of schizophrenic patients including symptom severity and functioning can be used for the description of the patient population. This approach could also be used for the assessment of treatment options in clinical practice.

## Competing interests

Joseph Peuskens has received honoraria as Consultant, Speaker, Advisory Board member and co-operation in clinical trials for Astra-Zeneca, Bristol-Myers Squibb, Eli Lilly, Janssen-Cilag, Lundbeck, and Pfizer.

Vibeke Porsdal, Yulia D'yachkova, Radim Brousil, and Walter Deberdt are employees of Eli Lilly and Company.

Jan Pecenak has received honoraria or sponsorship for conference attendance from Eli Lilly, Astra-Zeneca, Servier, Janssen-Cilag, and Lundbeck.

Peter Handest has received honoraria as speaker for Astra-Zeneca, Bristol-Myers Squibb, Eli Lilly and Janssen-Cilag.

## Authors’ Contributions

JP, JPE, PH, WD and VP have contributed to the design of the study and the interpretation of results. YD has participated in the design of the study and performed the statistical analysis. All authors read and approved the final manuscript.

## Pre-publication history

The pre-publication history for this paper can be accessed here:

http://www.biomedcentral.com/1471-244X/12/130/prepub
